# A Study to Determine How Frequently Patients Are Admitted to Acute General Hospitals With Psychiatric Presentations and Whether Acute Medics Feel Confident in Managing Such Cases

**DOI:** 10.1192/bjo.2024.521

**Published:** 2024-08-01

**Authors:** Ruby Viney, Katja Umla-Runge, Eleni Vrigkou

**Affiliations:** Cardiff University, Cardiff, United Kingdom

## Abstract

**Aims:**

Mental Health Trusts have seen significant funding cuts in recent years resulting in higher admissions to acute medical hospitals due to psychiatric disorders. Little information is available on the quantity of such presentations and no studies have explored how confident acute medical doctors feel in managing patients with mental health disorders.

**Primary objective:** To evaluate whether acute medical doctors feel confident in the common psychiatry topics required to manage patients presenting to medical hospitals with mental health disturbances.

**Secondary objective:** To determine how frequently patients with mental health disorders are admitted to medical beds, either primarily due to their psychiatric disorder or due to another medical problem.

**Methods:**

Acute medical doctors working in Merseyside, UK completed a self-report survey in which they rated their confidence level in relation to common psychiatric topics. Admission data for 4 large hospitals in Merseyside were analysed to determine the proportion of all patients admitted to medicine in a 1-year period who had a mental health disorder. Results were further broken down into primary diagnosis by ICD–11 code to determine which mental health conditions presented most frequently to general medical hospitals.

**Results:**

10 acute medical registrars and 33 acute medical consultants completed the survey. Most acute medical doctors felt at least partly confident in their psychiatry knowledge. However, around a quarter of doctors lacked confidence in managing psychotropic medications and performing risk assessments, with a third of acute doctors unsure how to access specialist psychiatric advice.

43.8% of all medical admissions had a mental health disorder. This was comprised of 3.1% who presented primarily due to a mental health illness, and 40.7% who had a mental health disorder but attended for a different reason. Substance misuse accounted for a significant proportion of these admissions.

**Conclusion:**

Despite almost half of patients admitted to medical beds experiencing mental illness, many acute medical doctors lack confidence managing psychiatric ailments and half of the respondents felt their medical training has not prepared them sufficiently.

In addition, many doctors are unsure how to access specialist advice when needed. This leaves both doctors and patients at risk of harm and suggests a need for additional psychiatric training for acute medical doctors and improved access to support.

